# Inactivating SARS-CoV-2 Surrogates on Surfaces Using Engineered Water Nanostructures Incorporated with Nature Derived Antimicrobials

**DOI:** 10.3390/nano12101735

**Published:** 2022-05-19

**Authors:** Nachiket Vaze, Anand R. Soorneedi, Matthew D. Moore, Philip Demokritou

**Affiliations:** 1 Center for Nanotechnology and Nanotoxicology, Department of Environmental Health, Harvard T. H. Chan School of Public Health, Harvard University, Boston, MA 02115, USA; nvaze@hsph.harvard.edu; 2 Department of Food Science, University of Massachusetts Amherst, Amherst, MA 01003, USA; asoorneedi@umass.edu (A.R.S.); mdmoore@foodsci.umass.edu (M.D.M.); 3 Nanoscience and Advanced Materials Research Center, Rutgers Biomedical Health Sciences, Rutgers University, Piscataway, NJ 08854, USA

**Keywords:** coronaviruses, nanotechnology, engineered nanomaterials, environmental health and safety

## Abstract

The continuing cases of COVID-19 due to emerging strains of the SARS-CoV-2 virus underscore the urgent need to develop effective antiviral technologies. A crucial aspect of reducing transmission of the virus is through environmental disinfection. To this end, a nanotechnology-based antimicrobial platform utilizing engineered water nanostructures (EWNS) was utilized to challenge the human coronavirus 229E (*HCoV-229E*), a surrogate of SARS-CoV-2, on surfaces. The EWNS were synthesized using electrospray and ionization of aqueous solutions of antimicrobials, had a size in the nanoscale, and contained both antimicrobial agents and reactive oxygen species (ROS). Various EWNS were synthesized using single active ingredients (AI) as well as their combinations. The results of EWNS treatment indicate that EWNS produced with a cocktail of hydrogen peroxide, citric acid, lysozyme, nisin, and triethylene glycol was able to inactivate 3.8 logs of *HCoV-229E*, in 30 s of treatment. The delivered dose of antimicrobials to the surface was measured to be in pico to nanograms. These results indicate the efficacy of EWNS technology as a nano-carrier for delivering a minuscule dose while inactivating *HCoV-229E*, making this an attractive technology against SARS-CoV-2.

## 1. Introduction

Despite the development of multiple effective vaccines, the COVID-19 pandemic caused by the SARS-CoV-2 coronavirus has not completely abated [1]. The goal of reaching herd immunity through a high percentage of vaccinated individuals has hit the barriers of vaccine hesitancy and the emergence of newer viral variants. These variants have led to the continuation of outbreaks globally [2]. Hence, arresting the spread of COVID-19 through environmental disinfection, by inactivating the SARS-CoV-2 virus, has become an important aspect of dealing with this global health problem [3].

Contaminated surfaces and inhalation of the airborne virus are the two modes of viral spread [4,5]. There are various technologies, such as ozone, ultraviolet light, acid fogs, etc., being researched for decontaminating surfaces by inactivating the virus [6,7]. These technologies cannot, however, be used for decontaminating sensitive surfaces such as food or be used in the presence of humans. For routine use by individuals, disinfectants such as bleach and alcohol-based products are widely used [8]. For skin disinfection, hand washing with soap and hand sanitizers is used. These methods have seen an increase in application since the beginning of the pandemic. This in turn has led to problems related to overuse [9].

The excessive use of bleach and alcohol-based products has also been shown to have a negative effect on the environment. Similarly, hand sanitizers and soaps can damage the sensitive human skin [10]. Other chemical compounds often used as disinfectants are hydrogen peroxide and quaternary ammonium (QAC). Hydrogen peroxide has limited applicability as a topical antiseptic and cannot be widely used due to its potential side effect such as itching, stinging, or swelling [11]. QACs have disadvantages, as they can leave toxic residues and lose effectiveness when mixed with organic matter [12].

Due to these factors, nanotechnology has emerged as an antimicrobial tool. Nanotechnology-based solutions have major advantages over conventional methods [13]. Engineered Nanomaterials (ENMs) such as polymeric nanoparticles, nanosilver, and magnetic nanoparticles have been used to inactivate various microorganisms, including coronaviruses. These ENMs have the advantages afforded by their nanoscale nature, such as a large surface-to-volume ratio, which enables them to be effective antiviral agents. However, there are several challenges to the application of ENMs, such as toxicity, low stability, and low yield [14]. This has led to the need to find safer nano alternatives.

The authors have developed a nano-carrier platform called Engineered Water Nanostructures (EWNS), which are aerosol nanodroplets synthesized using a combination of electrospray and ionization of an aqueous suspension of antimicrobials. These EWNS possess unique physicochemical properties, are highly mobile due to their nanoscale size, and remain airborne due to their high electric charge. They can interact with microorganisms on surfaces and in the air, delivering in a targeted and precise manner both reactive oxygen species (ROS) generated from ionization of water molecules and the antimicrobial ingredients used [15,16,17,18,19,20]. More recently the authors have successfully incorporated in the EWNS nature-derived antimicrobial active ingredients (AI) such as nisin, lysozyme, citric acid, hydrogen peroxide, as well as other chemicals which are being used in traditional so-called wet approaches (bulk application), such as quaternary ammonium, benzalkyl chloride, sodium hypochlorite [19]. These EWNS have been well characterized physiochemically and utilized as an antimicrobial tool in various applications in food and air disinfection and wound healing management. Our earlier studies have indicated the efficacy of these EWNS to inactivate bacteria on surfaces such as vegetables (spinach), skin (Porcine skin), and instrument surfaces (stainless steel) [16,17,20]. The EWNS have also been used to inactivate airborne viral particles of Influenza H1N1/PR8 [18] and *mycobacterium parafortuitum*, a surrogate of tuberculosis [21]. The remarkable fact is that in all these EWNS studies, the dose of the antimicrobial active ingredients delivered to the inoculated surface was minuscule (nanograms). This makes the EWNS a ‘dry’ method for the effective delivery of antimicrobials, as opposed to traditional ‘wet’ methods, where orders of magnitude higher dose of AIs is delivered in order to inactivate the microorganisms.

In this study, the authors have endeavored to study the ability of the EWNS to inactivate a surrogate of the SARS-CoV-2 virus, on surfaces. The human coronavirus 229E (*HCoV-229E*) was chosen as the surrogate of SARS-CoV-2. *HCoV-229E* is responsible for the common cold and has been utilized as a surrogate for studies to evaluate antimicrobial formulations to be used against other coronaviruses [22]. The *HCoV-229E* was inoculated onto a steel coupon surface and exposed to EWNS aerosol synthesized with various combinations of antimicrobials. The log reduction produced by each EWNS was calculated, to determine efficacy.

## 2. Materials and Methods

### 2.1. Generation of EWNS

**Figure 1:**
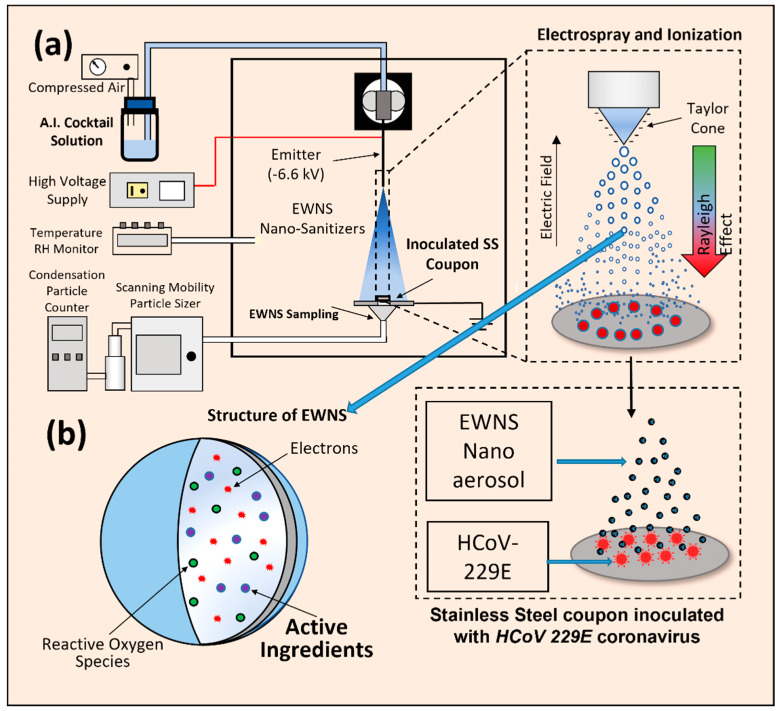
Detailed schematic to represent the generation of EWNS and the treatment of *HCoV-229E* inoculated surface (**a**). The structure of an individual EWNS (**b**) containing the A.I., ROS and charges is also shown.

The concept of EWNS generation is illustrated in Figure 1. The main process involved in the generation of EWNS is a combined electrospray-ionization process, detailed in an earlier publication by the group [15]. To expound, a stainless-steel capillary (EWNS emitter) is held vertically, and a funnel-shaped ground electrode assembly is placed directly underneath. The EWNS emitter is connected, at the top, to a container containing the AI solution. This container is connected to an air compressor, which is used to push the liquid flow through the emitter. A high voltage power supply (Spraybase, Cambridge, MA, USA) is utilized to produce the −6.8 kV to be delivered to the emitter. The emitter is held at a distance of 4 cm from the ground electrode. The grounded electrode is a disk that sits atop a funnel that is connected to a sampling apparatus that can be used to draw in the EWNS nanodroplets for characterization. During the process of EWNS generation, the applied electric field causes, at the tip of the capillary, the generation of the so-called Taylor cone, and, from the tip of this cone, highly charged nanodroplets, of the aqueous suspension of various AIs, are emitted. This Taylor cone is monitored visually using a camera. The shape and stability of this Taylor cone are monitored with the camera. Once a stable cone is confirmed, the EWNS nanodroplets are then sampled with the apparatus described above, to confirm their generation.

### 2.2. Selection of Antimicrobial Active Ingredients

From the peroxide class, hydrogen peroxide was chosen, as it is widely being used in vapor form as a disinfectant in healthcare settings. An organic acid, citric acid (CA), was chosen as an antimicrobial from nature. CA is found in various citrus fruits and has antibacterial effects. Other natural substances explored were an enzyme (lysozyme) and a peptide (nisin), both currently being researched for their efficacy against bacteria and viruses. Lysozyme is found in bodily fluids such as tears, whereas nisin is used frequently in the food preservation industry [23,24]. In addition to these, triethylene glycol (TEG) was evaluated for the first time with the EWNS system. A recent study has shown that atomized TEG is effective in inactivating common nosocomial pathogens [25]. This has made it an attractive AI candidate to be utilized in the EWNS system.

### 2.3. Physicochemical Characterization of EWNS

The aerosol size and concentration of various EWNS produced was assessed with a Scanning Mobility Particle Sizer (SMPS) (TSI, Shoreview, MN, USA). The SMPS gives a size distribution of the nano-aerosol measured, and the arithmetic mean diameter of each EWNS formulation was obtained using the Aerosol Instrument Manager software (TSI, Shoreview, MN, USA). The nanodroplet concentration of the EWNS was also reported. Ten discreet measurements were performed, each for a duration of 120 s, and the average of the measurements was reported. The total dose of each EWNS treatment was obtained by calculating the mass of the nanodroplets produced during one minute of EWNS generation.

### 2.4. Inoculation of HCoV-229E and Recovery

The human coronavirus 229E (ATCC^®^ VR-740™) was used at a stock concentration of 10^7^ pfu/mL. 10 µL of the virus stock suspension (~10^5^ µL) was inoculated as equally sized droplets on the surface of previously sterilized stainless-steel coupons (18.2 mm diameter). The coupons were placed in sterile Petri dishes. The coupons were then allowed to air dry till the droplets were visibly dry. After the drying step, the coupons were removed for EWNS treatment.

### 2.5. EWNS Treatment

The coupons were placed underneath the EWNS emitter, as shown in Figure 1a, and EWNS treatment was carried out for the specified duration of treatment. Post treatment, 100 µL of DMEM complete growth medium (10% FBS and 1% Penicillin-Streptomycin) was gently added to the surface of the coupons. The surface was gently rinsed by pipetting the medium up and down. The rinsate was collected and used for preparing serial dilutions of the recovered virus in DMEM complete growth medium. The serially diluted virus suspension was used for setting up a viral plaque assay. Control coupons without EWNS treatment were also included in triplicate in this study.

### 2.6. HCoV-229E Plaque Assay

Briefly, Huh 7.5 cells were plated in 12-well plates and incubated overnight at 37 °C (5% CO_2_). The following day, virus suspension (10^7^ pfu/mL) is serially diluted in DMEM complete growth medium (10% FBS and 1% Penicillin-Streptomycin). After aspirating the media from each well, 100 ul of virus dilutions were added to each well. The plates are incubated for 1 h at 37 °C with 5% CO_2_ with gentle rocking every 10 min. Following incubation, a mixture of 2X DMEM and 2.4% Avicel is overlayed on the cells avoiding any air bubbles. The cells along with the virus and Avicel overlay were incubated in a 37 °C incubator (5% CO_2_) for 4 days. For visualizing plaques after the 4-day incubation, cells were fixed with 5% formaldehyde for 1 h at room temperature. Formaldehyde was aspirated into an appropriate chemical waste container and the cell monolayer was gently rinsed with tap water or PBS. The cells were stained with 2% crystal violet solution for 30–60 min at RT on a shaking platform. The cells were then rinsed with tap water or PBS. We then let the monolayer air dry for 15–20 min inside the biosafety cabinet. The plaques were counted, and the pfu/mL was calculated using the following formula: Plaque count X virus dilution X 10 = pfu/mL sample.

### 2.7. Statistical Analysis

Each EWNS treatment was performed in triplicate. Each data point represents the calculated arithmetic mean of three replicates. The error bars represent the standard deviation between the three replicates.

## 3. Results and Discussion

### Generation and Physicochemical Characterization of EWNS Nanoaerosol

The EWNS were generated according to a well-established method, quoted in detail in the Materials and Methods section and illustrated in Figure 1. A detailed assessment of the incorporation of AIs across various antimicrobial classes into EWNS has been conducted in an earlier study by the authors. AIs were chosen to cover a broad range of antimicrobials. The selection logic for these AIs is detailed in the Materials and Methods section. These AIs are detailed in Table 1. 

**Figure 2:**
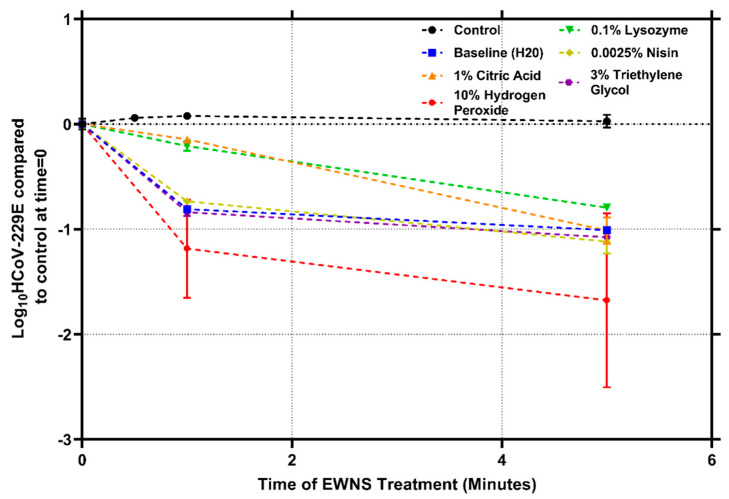
Inactivation of *HCoV-229E* on surface, after treatment with EWNS. The active ingredient utilized for producing each EWNS for treatment is indicated. Error bars represent standard deviation.

The dose rate was calculated using a number of nanodroplets and is detailed as well. The “baseline” EWNS were synthesized using only water (no antimicrobials) and had a diameter of 18.28 (±1.32) nm. This size is similar to what has been observed in earlier studies [15]. The 10% H_2_O_2_ produced EWNS with a size of 10.62 (±2.15) nm. Citric acid (CA) produced larger EWNS nanostructures with a diameter of 35.6 (±1.1) nm. This is consistent with earlier studies with EWNS, where CA nanodroplets have been shown to have a larger diameter [19]. Lysozyme and nisin produced EWNS that were smaller in diameter with 20.05 (±0.61) and 14.3 (±0.5) nm, respectively. The largest EWNS created with a single AI was seen with the TEG. The diameter of the TEG EWNS nanodroplet was 56.58 (±8.04) nm. The efficacy of each EWNS in terms of inactivating the virus inoculated on coupons was assessed. For EWNS generated with single AIs, the results are summarized in Figure 2. The no exposure (control) shows no decay after 5 min of being inoculated onto the coupons. This is congruous with studies that have indicated the high survivability of *HCoV-229E* on surfaces over time. *HCoV-229E* has been shown to stay active on stainless steel surfaces for up to 5 days [22]. Because of this high survivability, there is a substantial risk of transmission through fomites and inactivating *HCoV-229E* on surfaces is a challenging task. For the treatment with baseline EWNS produced with water (baseline EWNS), 0.81 (±0.025) log reduction was observed after 1 min and 1 (±0.02) log after 5 min. The inactivation rate observed here is similar to the results obtained with the baseline EWNS against Influenza H1N1/PR/8 [18]. For citric acid and lysozyme-produced EWNS nano-sanitizers, the inactivation showed a linear inactivation curve, with 1.01 (±0.12) and 0.79 (±0.009) logs inactivation at 5 min, respectively. The calculated dose of the citric acid and lysozyme for the 5-min exposure was minuscule, 117.81 (±29.13) and 0.83 (±0.3) picogram, respectively. These results indicate that the incorporation of lysozyme into EWNS leads to efficacious inactivation with a picogram dose. For 1 min of nisin-EWNS treatment led to 0.73 (±0.02) log inactivation. For 5 min treatment, the inactivation was 1.11 (±0.11) logs with the calculated delivered dose of nisin being 0.07 (±0.01) picogram for the 5-min treatment. Nisin has been investigated for its ability to bind with the ACE2 receptor of the coronavirus and its potential to be an anti-coronavirus agent has been proposed. Evidence from this result suggests that EWNS is an effective nano-carrier for the potential use of nisin in this capacity [26]. For the case of EWNS nano-sanitizers produced with 10% H_2_O_2_, the inactivation at 1 and 5 min was 1.18 (±0.46) logs and 1.67 (±0.82) logs respectively, with the calculated dose being 0.32 (±0.12) picogram for 5 min treatment. Compared to other AIs evaluated, there is greater variability observed here between the three treatment runs. These results indicate the efficacy of the H_2_O_2_ EWNS treatment. Recent studies into the efficiency of H_2_O_2_ against *HCoV-229E* have shown that, although a bulk application of 0.5% H_2_O_2_ solution was effective against *HCoV-229E*, the potential cellular toxicity of the solution needed to be mitigated before in vivo application [27]. For skin disinfection, a 3% solution of hydrogen peroxide is commonly used, but it also can have deleterious effects on the skin leading to serious skin issues [28]. The picogram level dose delivered for effective inactivation suggests that the EWNS platform could make H_2_O_2_ applicable in these settings, where it cannot be applied due to toxicity and safety concerns. It is worth mentioning, however, that the observed large SD for the inactivation produced here is because the inactivation depends on the dose of the active ingredient delivered to the viral inoculum. As the total dose of each EWNS treatment was obtained by calculating the mass of the nanodroplets produced during one minute of EWNS generation, a large deviation in nanodroplet size as compared to the mean would lead to a large deviation in volume, and thus, mass. Hence, for H_2_O_2_ as the active ingredient, the generated EWNS nanoaerosol has a large deviation in the size distribution of the nanodroplets generated which results in a large deviation in the delivered dose and inactivation efficacy. For TEG, a similar trend of inactivation was observed as H_2_O_2_ and nisin, with 0.83 (±0.03) log in 1 min and 1.07 (±0.01) log in 5 min of treatment, with the dose of TEG for 5 min exposure being 161.87 (±56.78) picograms.

In summary, the EWNS nano-sanitizers produced with various AIs were able to significantly inactivate *HCoV-229E* on a surface by delivering only minuscule levels of AIs. At 5 min of treatment, no significant difference was observed for all five AIs evaluated. H_2_O_2_ produced the highest inactivation level. H_2_O_2_, TEG, and nisin produced a biphasic inactivation, which has been observed in many earlier studies with antimicrobial efficacy testing [29]. When compared to the baseline EWNS, the results were not significantly improved after the addition of individual AIs. Another observation is the fact that dose values were higher for AIs that produced larger EWNS nanodroplets, namely citric acid, and TEG.

Earlier studies with AI-based EWNS have shown that these cocktail EWNS nanodroplets significantly increase the inactivation efficacy, as compared to single AIs due to synergistic effects. For example, EWNS produced with a cocktail of 1%H_2_O_2_ and 1%CA were utilized to inactivate *E. coli* on surfaces and the results of the study indicated that the efficacy rate of the cocktail was higher than that of the individual active ingredients combined [17]. In another study conducted by the authors for assessing the efficacy of EWNS against pathogens relevant to hand hygiene, this concept was further expanded with more AIs and their combinations were studied. A cocktail was developed, containing 10% H_2_O_2_, 1% CA, 0.1% lysozyme, and 0.0025% nisin, and it was found to have produced a significant reduction in the concentration of non-enveloped phage MS2 [19].

**Figure 3:**
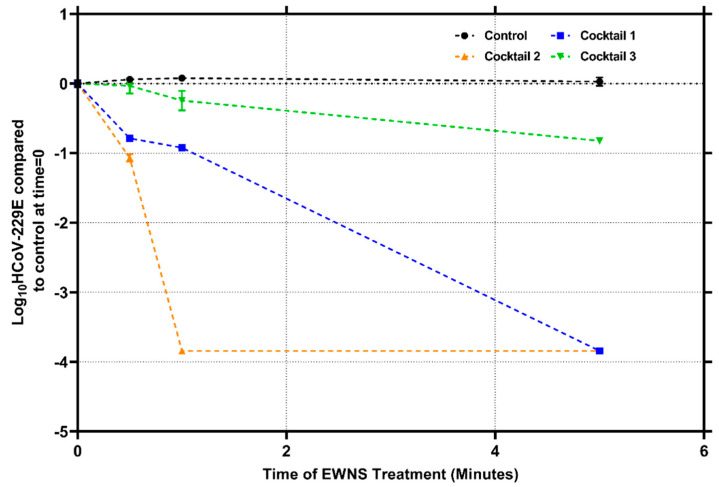
Inactivation of *HCoV-229E* on surface, after treatment with EWNS. The active ingredient cocktail utilized for producing each EWNS for treatment is indicated. Error bars represent standard deviation.

The same cocktail used in the hand hygiene study [19] was utilized in this study to produce EWNS nano-sanitizers and its efficacy against the *HCoV-229E* was assessed (cocktail 1). The results of the inactivation produced are shown in Figure 3. The EWNS produced by this cocktail were found to have an average diameter size of 24.85 (±3.75) nm. Their inactivation observed for 30 s and 1 min was 0.78 (±0.017) and 0.92 (±0.012) logs. However, at 5 min treatment, complete inactivation, which corresponded to 3.8 logs reduction, was observed. The calculated dose delivered to the viral inoculum for 5 min exposure was 168.09 (±76.50) picograms. These results demonstrate the efficacy of the cocktail EWNS to inactivate *HCoV-229E*, with a minuscule dose of the antiviral active ingredients utilized. The picogram dosage also indicates the precisely targeted delivery of the active ingredients to the viral particles.

Furthermore, triethylene glycol (TEG), was added to the cocktail to further assess antiviral efficacy, the AI cocktail which contains 10% H_2_O_2_, 1% CA, 0.1% lysozyme, and 0.0025% nisin and 3% TEG was used to generate EWNS nano-sanitizers (cocktail 2). The size measurement shows that these nanodroplets were 42.71 (±3.36) nm in average size. This indicates that the addition of TEG led to an increase in the size of the EWNS produced. This would correspond with the observation that TEG as a single AI, produced the largest EWNS nanodroplet observed in this study at 56.58 (±8.04) nm. More interestingly, the addition to the AI cocktail of TEG resulted in an increase in the antiviral efficacy of the generated EWNS nano-sanitizers. Here, 30 s of exposure led to a 1.06 (±0.05) logs reduction. For 1 min treatment, complete inactivation was observed, with a 3.8 logs reduction. Further timepoint was assessed at 5 min of treatment. The 3.8 logs reduction was also observed for 5 min. These results indicate an increase in the inactivation efficacy after the addition of TEG. The calculated dose delivered to the viral inoculum for complete inactivation in 1 min of exposure was 124.67 (±53.77) picograms. Another cocktail, containing only 10% H_2_O_2_, 1% CA, 0.0025% nisin, and 3% TEG was used, without any lysozyme added (cocktail 3). For this cocktail, the size characterization indicated the generation of a smaller nanodroplet than the other two cocktails utilized, with a 17.76 (±0.41) nm size. This is interesting as it shows the influence of lysozyme on the size of the nanodroplets produced. This new cocktail of EWNS nanodroplets was challenged with *HCoV-229E*. The results showed only a 0.24 (±0.14) logs reduction after 1 min treatment, which increased to 0.82 (±0.046) logs for 5 min of treatment. The total dose of the EWNS to the viral inoculum was 65.13 (±20.10) picogram.

The EWNS generation process involves only water and the minuscule amounts of active ingredients utilized with no chemical residues or by-products left behind. Our previous publications address this issue in great detail [17]. It is worth emphasizing that the active ingredients utilized and delivered using the EWNS nanocarrier platform are nontoxic and nature-derived, and only minuscule amounts are delivered (nanogram levels).

It is also worth noting that although the EWNS technology has been shown to produce significant inactivation in the viral inoculum, there are, however, certain limitations of the methodology utilized. The *HCoV-229E* virus was inoculated onto the coupons in a salt-rich solution (4% FBS + DMEM). This makes it more challenging for the EWNS technology to reach the viral cells amidst the salt deposits, reducing its efficacy of inactivation. There is also a limitation to the recovery of exposed inoculum, however, the authors have attempted to maximize the recovery to obtain a more accurate picture of the EWNS’ effect on the virus. In addition, the inactivation results are based on surface inoculation rather than the virus suspended in the air. Future studies will focus on the ability of the EWNS nanoaerosol to interact with the virus in the air and provide efficient inactivation.

## 4. Conclusions

In summary, in this study, we have evaluated the SARS-CoV-2 surrogate antiviral efficacy of the EWNS platform using antimicrobials and their mixtures. The results with the AI cocktail have indicated the efficacy of this platform to inactivate *HCoV-229E* on surfaces. Compared to conventional application practices for the AIs utilized in this study, the EWNS nano-carrier platform is advantageous, as it requires minuscule amounts of AIs for effective inactivation and the delivery is performed via aerosol. The targeted and precise delivery of the nanodroplets makes this technology an alternative to conventional (wet) treatments. Further research into the application of EWNS for air disinfection is warranted given the promising data on surface inactivation.

## Figures and Tables

**Table 1:** Active Ingredients (AIs) utilized to generate various EWNS.

Active Ingredients (Concentrations)	Size (nm)	Nanodroplet Concentration (#/cc)	Dose Rate (pg/min)
Baseline (Water, No A.I. added)	18.28 ± 1.32	96,189 ± 16,720	NA
Hydrogen peroxide (10% *w*/*v*)	10.62 ± 2.15	2042 ± 779	0.06 ± 0.02
Citric acid (1% *w*/*v*)	35.6 ± 1.1	199,578 ± 49,343	23.56 ± 5.83
0.1% Lysozyme (0.1% *w*/*v*)	20.05 ± 0.61	79,006 ± 28,000	0.17 ± 0.06
0.0025% Nisin (0.0025 *w*/*v*)	14.3 ± 0.5	701,301 ± 55,276	0.01
3% Triethylene glycol (3% *w*/*v*)	56.58 ± 8.04	22,769 ± 7987	32.37 ± 11.36
Hydrogen peroxide (10% *w*/*v*) + Citric acid (1% *w*/*v*) + Lysozyme (0.1% *w*/*v*) + Nisin (0.0025% *w*/*v*) (Cocktail 1)	24.85 ± 3.75	75,409 ± 34,320	33.62 ± 15.30
Hydrogen peroxide (10% *w*/*v*) + Citric acid (1% *w*/*v*) + Lysozyme (0.1% *w*/*v*) + Nisin (0.0025% *w*/*v*) + Triethylene glycol (3% *w*/*v*) (Cocktail 2)	42.71 ± 3.36	43,364 ± 18,702	124.67 ± 53.77
Hydrogen peroxide (10% *w*/*v*) + Citric acid (1% *w*/*v*) + Nisin (0.0025% *w*/*v*) + Triethylene glycol (3% *w*/*v*) (Cocktail 3)	17.76 ± 0.41	63,463 ± 19,586	13.03 ± 4.02

## Data Availability

All data generated or analyzed during this study are included.
